# Role of biochar, compost and plant growth promoting rhizobacteria in the management of tomato early blight disease

**DOI:** 10.1038/s41598-021-85633-4

**Published:** 2021-03-17

**Authors:** Mujahid Rasool, Adnan Akhter, Gerhard Soja, Muhammad Saleem Haider

**Affiliations:** 1 Faculty of Agricultural Sciences, Department of Plant Pathology, University of the Punjab, P.O Box: 54590, Lahore, Pakistan; 2 Department of Health and Environment, Austrian Institute of Technology, Tulln, Austria; 3 Institute of Chemical and Energy Engineering, University for Natural Resources and Life Sciences, Muthgasse 107, 1190 Vienna, Austria

**Keywords:** Plant sciences, Pathogens

## Abstract

The individual role of biochar, compost and PGPR has been widely studied in increasing the productivity of plants by inducing resistance against phyto-pathogens. However, the knowledge on combined effect of biochar and PGPR on plant health and management of foliar pathogens is still at juvenile stage. The effect of green waste biochar (GWB) and wood biochar (WB), together with compost (Comp) and plant growth promoting rhizobacteria (PGPR; *Bacillus subtilis*) was examined on tomato (*Solanum lycopersicum* L.) physiology and *Alternaria solani* development both in vivo and in vitro. Tomato plants were raised in potting mixture modified with only compost (Comp) at application rate of 20% (v/v), and along with WB and GWB at application rate of 3 and 6% (v/v), each separately, in combination with or without *B. subtilis*. In comparison with WB amended soil substrate, percentage disease index was significantly reduced in GWB amended treatments (Comp + 6%GWB and Comp + 3%GWB; 48.21 and 35.6%, respectively). Whereas, in the presence of *B. subtilis* disease suppression was also maximum (up to 80%) in the substrate containing GWB. Tomato plant growth and physiological parameters were significantly higher in treatment containing GWB (6%) alone as well as in combination with PGPR. *Alternaria solani* mycelial growth inhibition was less than 50% in comp, WB and GWB amended growth media, whereas *B. subtilis* induced maximum inhibition (55.75%). Conclusively, the variable impact of WB, GWB and subsequently their concentrations in the soil substrate was evident on early blight development and plant physiology. To our knowledge, this is the first report implying biochar in synergism with PGPR to hinder the early blight development in tomatoes.

## Introduction

Tomato (*Solanum lycopersicum* L.) is an extensively cultivated horticultural crop, with global consumption of second to potato^1^. In 2018, around 182 million tons of tomato has been produced on an area of 4.76 million hectares in more than 150 countries^2^. Tomato is the richest source of vitamins (A and C) and antioxidant (lycopene pigment) making it an integral component of our balanced diet^3^. Early blight (EB) on tomato caused by *Alternaria solani*, is an air-borne soil inhabiting fungus with the reputation of being one of the most destructive disease of tomato solely accounting for yield losses of up to 80%^4^. Disease symptoms on tomatoes include small dark brown bullseye spots with concentric ring patterns, which become enlarged with the progression of infection and cover the whole leaf^5^. The pathogen can overwinter in plant debris or soil as conidia or mycelia and becomes a source of inoculum upon availability of suitable temperature (27–32 °C), humidity (50–70%) and host plant^6^. For the management of EB disease of tomato, many techniques have been in used such as chemical control by using fungicides e.g. propineb, mancozeb, copper oxychlorode, Tebuconazole, propiconazole^7,8^ and selection of resistant genotypes^9–12^. In addition, different bio-control techniques are also being employed for the management of EB such as PGPR-mediated protection by stimulating production and activity of antioxidant peroxidase (POX) and polyphenol oxidase (PPO) enzymes in host plants^4,13^, use of galrlic (*Allium sativum)* extract^12^, essential oils extracted from different varieties of Eucalyptus^14^, nano-particles biosynthesized from fruit peel extract of citrus kinnow^15^, extract from wild medicinal plants including *Calotropis procera* (*Aitón*) W. T. Aiton^16^ and *Putranjiva roxburghii*^17^*.* Besides different control strategies, chemical control by fungicides has been regarded asa predominant practice for the EB management^18^. Agro-chemicals, in addition to causing severe damages to human and environment health are also responsible for the development of resistance in *A. solani* against different fungicides^19^. Therefore, we need to explore chemicals independent, environment friendly organic solutions for the management of *A. solani* in tomato.

Amongst the innovative and novel organic materials, biochar a charcoal like product formed by pyrolysis (a process involving heating of organic materials in an oxygen deficient environment) has shown promises against many plant pathogens^20^. The physico-chemical properties of biochars are dynamic in nature, dependent upon source of raw organic material (e.g., green waste, wood chips, crop residues, poultry manure etc.) as well as processing conditions especially the temperature of pyrolysis^21–23^. Recent studies have revealed that the application of biochar in combination with compost has synergistic effects on growth and nutrient uptake by plants^24,25^.

Additionally, biochar has been reported to be effective in suppressing diseases caused by both soil-borne and air-borne plant pathogens such as *Fusarium oxysporum* f. sp. *lycopersici* on tomato^26^, *Rhizoctonia solani* on cucumber^27^, while *Podosphaera aphanis* on strawberry^28^, *Botrytis cinerea*, *Leveillula taurica* on tomato and pepper^29,30^. However, the effect of biochar as soil amendment on *A. solani* causing EB of tomato, a pathosystem of huge economic impact, is yet to be determined. In horticulture practices, compost has been used to improve crop yield and quality of soil^31^. As it’s a rich source of nutrients e.g. P and N, thus reduce the need for application of inorganic fertilizers^32^. The properties of compost rely on various factors such as composting conditions, originating feedstock such as plant-green-waste^33^, or animal source such as sheep manure^34^ and poultry residues^35^. Further, most of the published studies report that compost amendments has ability to suppress the most common air-borne diseases of tomato plant including EB (*A. solani*)^36–38^ and septoria blight (*Septoria lycopersici*)^39–41^.

Moreover, it has also been proposed that the combination of biochar and compost induce modifications in physical and chemical properties of soil, leading to better plant growth and production^42–45^. There was synergistic impact of co-application of biochar and compost for the management of soil-borne diseases and enhancing the activity of beneficial microbial populations of the soil including arbuscular mycorrhizal fungi^46^, plant growth promoting rhizobacteria (PGPR) and other bio-control agents^47^. Among diverse microbial communities of soil, bacteria including PGPR outnumber all others. PGPR like *Bacillus subtilis*, *Pseudomonas fluorescens*, *Burkholderia phytofirmans* and *Azospirillum* spp. not only improves nutrient access to plants but also suppress diseases and other abiotic stresses faced by the plants^48–50^. PGPR suppress foliar pathogens by inducing systemic resistance via metabolic pathways involving ethylene or jasmonic acid (JA)^51,52^. Therefore, considering a balanced use of soil organic additives and biological antagonists provide an innovative platform to control the soil-borne as well as aerial pathogens^53^.

The individual role of biochar, compost and PGPR against foliar disease suppression has been well documented^30,37,38,52^. While, synergistic potential of biochar and PGPR combination in plant growth promotion has only been studied in few times such as in soybean^54^, French beans (*Phaseolus vulgaris*)^55^, chickpea (*Cicer arietinum*)^56^ and wheat^57^. Further, Hafez et al.^58^ and Danish et al.^59^ studied the combined effect of biochar and PGPR on rice (*Oryza sativa* L.) and maize (*Zea mays* L.) for the management of abiotic stresses such as salinity and drought, respectively. In this study we focused on economically important pathosystem, comprising of tomato with annual production value of ∼$59 billion and *A. solani* causing enormous losses both in the field and greenhouse^5^. Therefore, the present study was designed to achieve the following objectives, (a) to assess the influence of biochars made from different feed stocks i.e. WB and GWB, when applied at different concentrations to the soil substrate on tomato growth and on the development of *A. solani*, (b) to evaluate combined impact of PGPR (*B. subtilis*), biochar and compost on physiological growth parameters and suppression of EB of tomato, and, (c) to evaluate the in vitro antifungal potential of biochars, compost and PGPR against *A. solani* mycelial growth. It is expected that outcome of the study will provide a way forward in plant disease management by organic innovations, while fulfilling the objectives of sustainable agricultural practices.

## Results

### Molecular analysis for the confirmation of the *Alternaria solani*

The ITS and β-tubulin 1 gene primers amplified PCR products of 580 bp and 364 bp, respectively (Supplementary Fig. S1). The sequences of the PCR products were deposited to the Genbank and received accession numbers MT899419 and MT899420 for ITS and β-tubulin 1, respectively. BLASTn comparison analysis of the ITS GenBank accession No. MT899419 has shown close homology (99.66%) with the *A. solani* isolates from china (MG012294.1 and MG012293.1), while β-tubulin 1 GenBank accession No. MT899420 has similarity (99.45%) with *A. solani* isolate from Korea (JF417707.1).

### Estimation of plant growth parameters

**Figure 1:**
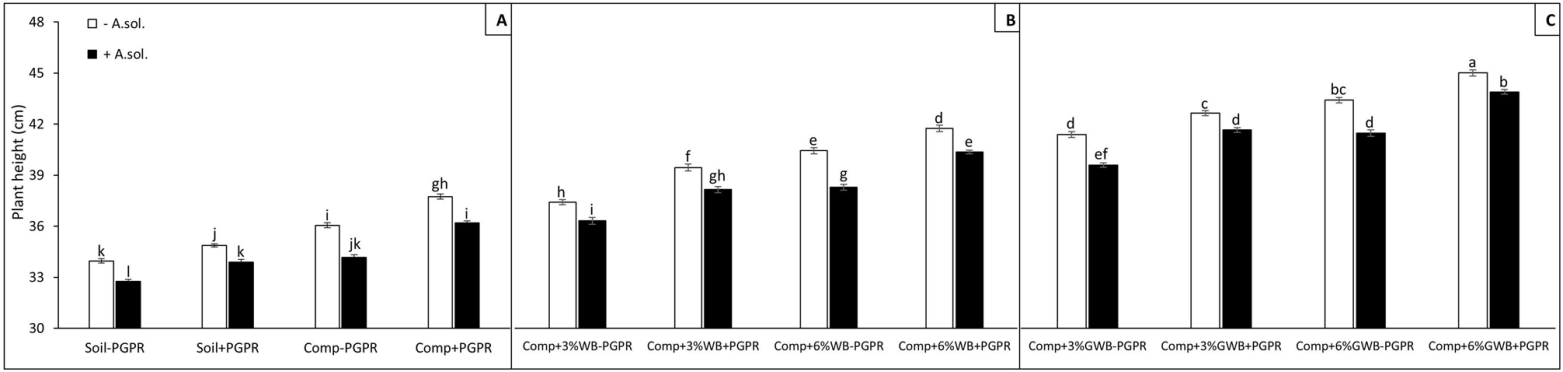
Effect of *Alternaria solani* and PGPR on tomato plant height grown in various soil substrate compositions including; (**A**) soil and compost (Comp) alone with (+ PGPR) and without PGPR (− PGPR), inoculated (+ *A. solani*) or un-inoculated (− *A. solani*), (**B**) compost (Comp) with 3 and 6% wood biochar (WB) with (+ PGPR) and without PGPR (− PGPR), inoculated (+ *A. solani*) or un-inoculated (− *A. solani*), (**C**) compost (Comp) with 3 and 6% Green waste biochar (GWB) with (+ PGPR) and without PGPR (− PGPR), inoculated (+ *A. solani*) or un-inoculated (− *A. solani*). All values represent mean ± SE, recorded 40 days after transplantation. Bars with different letters on the top suggest significant difference as per Tukey’s HSD test (*P* ≤ 0.05).

The reduction in shoot height was significant among all the *A. solani* inoculated treatments (Fig. 1A–C). Maximum plant height (45.21 and 44.07 cm) was in treatment ‘Comp + 6%GWB + PGPR’ in the absence and presence of disease stress, respectively (Fig. 1C). Amongst *A. solani* inoculated plants, significant reduction in shoot heights were ranked as (according to soil amendment; from minimum reduction to maximum) green waste biochar amended treatments were followed by the wood biochar, compost and lastly by the un-amended soil control both with and without the PGPR.

**Figure 2:**
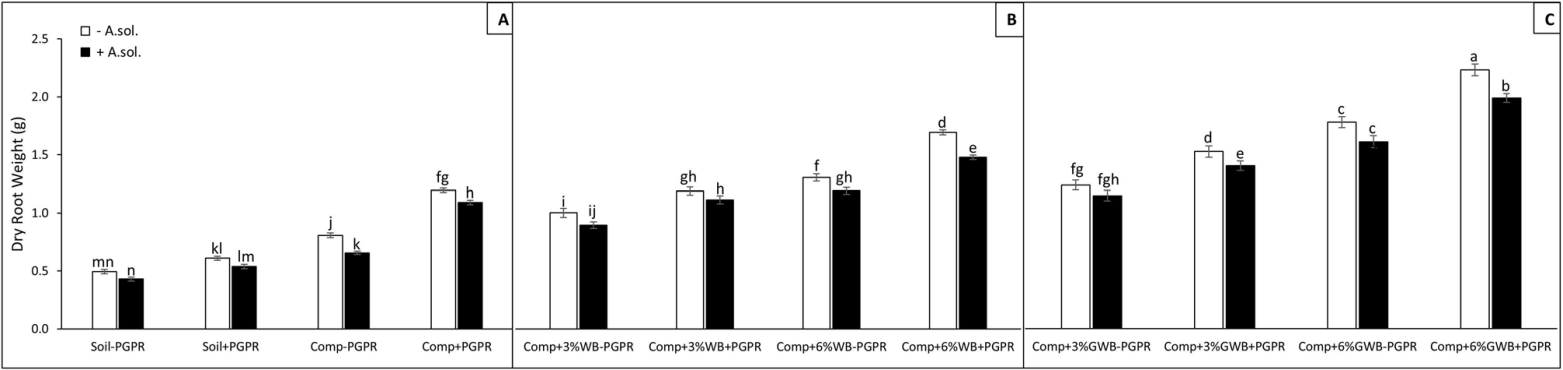
Effect of *Alternaria solani* and PGPR on dry root weight of tomato raised in various soil substrate compositions including; (**A**) soil and compost (Comp) alone with (+ PGPR) and without PGPR (− PGPR), inoculated (+ *A. solani*) or un-inoculated (− *A. solani*), (**B**) compost (Comp) with 3 and 6% wood biochar (WB) with (+ PGPR) and without PGPR (− PGPR), inoculated (+ *A. solani*) or un-inoculated (− *A. solani*), (**C**) compost (Comp) with 3 and 6% Green waste biochar (GWB) with (+ PGPR) and without PGPR (− PGPR), inoculated (+ *A. solani*) or un-inoculated (− *A. solani*). All values represent mean ± SE, recorded 40 days after transplantation. Bars with different letters on the top suggest significant difference as per Tukey’s HSD test (*P* ≤ 0.05).

**Figure 3:**
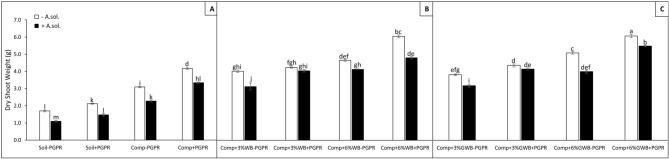
Effect of *Alternaria solani* and PGPR on dry shoot weight of tomato raised in various soil substrate compositions including; (**A**) soil and compost (Comp) alone with (+ PGPR) and without PGPR (− PGPR), inoculated (+ *A. solani*) or un-inoculated (− *A. solani*), (**B**) compost (Comp) with 3 and 6% wood biochar (WB) with (+ PGPR) and without PGPR (− PGPR), inoculated (+ *A. solani*) or un-inoculated (− *A. solani*), (**C**) compost (Comp) with 3 and 6% Green waste biochar (GWB) with (+ PGPR) and without PGPR (− PGPR), inoculated (+ *A. solani*) or un-inoculated (− *A. solani*). All values represent mean ± SE, recorded 40 days after transplantation. Bars with different letters on the top suggest significant difference as per Tukey’s HSD test (*P* ≤ 0.05).

The results of three-way ANOVA presented as *P* values are summarized in Table 1. Soil substrate compositions comprising of compost alone and in combination with 3 and 6% of each wood biochar and green waste biochar, PGPR and *A. solani* served as main factors. Plant height was significantly (*P* < 0.001) influenced by the interactive effect of soil substrate composition (SC) with both PGPR and *A. solani* (AS) [(SC × PGPR and SC × AS, respectively)], as well as by the interaction of PGPR and *A. solani* (PGPR × AS; (*P* ≤ 0.05). Therefore, in the presence of PGPR, both wood and green waste biochar had a positive impact on plant height, root and shoot dry weight (Figs. 1, 2, 3). Among all the treatments either with or without biochar, *A. solani* inoculation caused reduction in dry weights of above and below ground plant parts (Figs. 2, 3). However, maximum root dry weight (2.22 g) was found in tomato plants grown in treatment ‘Comp + 6%GWB + PGPR’, while no significant reduction recorded in dry root biomass under early blight influence (Comp + 6%GWB + PGPR + *A. sol*; 1.97 g). There was an increase of ~ 23% in root dry biomass of + *A. solani* tomato plants grown in ‘Comp + 6%WB + PGPR’ and ‘Comp + 6%GWB + PGPR’ substrates unlike their − PGPR counterparts (Fig. 2B,C). In addition to the significant (*P* < 0.001) effect of individual variables, there was also a significant (*P* ≤ 0.05) interaction effect between soil composition and *A. solani* (SC × AS) on dry root weight. While, in case of dry shoot weight of tomato plants a significant (*P* < 0.001) three way interaction between SC × PGPR × AS was reported (Table 1).

**Table 1:** Three-way ANOVA results represented as level of significance of the effect of factors soil substrate composition (SC), *Alternaria solani* (AS) and plant growth promoting rhizobacteria; *Bacillus subtilis* (BS) and their interactions on tomato plant growth and physiological parameters.

	Plant height	Dry root weight	Dry shoot weight	Nitrogen contents	Phosphorus contents	Potassium contents	Chlorophyll contents
SC	***	***	***	***	***	***	***
PGPR	***	***	***	***	***	***	***
AS	***	***	***	***	***	***	***
SC × PGPR	***	***	***	ns	***	ns	**
SC × AS	*	*	***	ns	***	**	ns
PGPR × AS	***	ns	***	ns	ns	ns	ns
SC × AS × PGPR	ns	ns	***	ns	ns	ns	ns

*ns* non-significant.

*P ≤ 0.05; **P < 0.01 ***P < 0.001.

Among compost and biochar amended treatments, lowest dry shoot weight (2.73 g) was in the ‘Comp + 3%WB − PGPR’ treatment with *A. solani* induced disease stress, and highest (5.92 g) in ‘Comp + 6%GWB + PGPR’ treatment without *A. solani* (Fig. 3B,C). The pathogen (*A. solani*) induced reduction in the shoot dry weight was significant in all the treatments, however, only found to be non-significant in 3%GWB amended treatment (Comp + 3%GWB + PGPR) in comparison to its un-inoculated compliment. In the presence of disease stress, maximum shoot dry biomass (5.04 g) was measured in plants grown in ‘Comp + 6%GWB + PGPR’ treatment, while the lowest (1.09 g) was in soil control without any organic amendments and PGPR (Fig. 3A,C). The maximum reduction 26.45% and 20.86% in shoot dry weight was recorded in treatment ‘Comp − PGPR’ and ‘Comp + 6%WB + PGPR’, respectively in comparison to their respective *A. solani* free counterparts (Fig. 3A,B).

### Plant physiological parameters

#### Estimation of chlorophyll contents of tomato plants

In addition to the interactive effect of SC × PGPR (*P* < 0.01), all main factors including soil composition, PGPR and *A. solani* significantly (*P* < 0.001) influenced the chlorophyll contents of tomato plants. The soil composition containing 6%GWB significantly increased the quantity of chlorophyll in tomato plants as described in Table 2. Maximum contents of chlorophyll 44.02 ± 0.24 and 43.07 ± 0.08 were observed in plants raised in ‘Comp + 6%GWB + PGPR’ and ‘Comp + 6%GWB − PGPR’ treatments, respectively in the absence of *A. solani*. Overall, *A. solani* inoculation induced reduction in the chlorophyll contents. While, the plants inoculated with *A. solani*, grown in compost and/or biochar amended treatments irrespective to the concentration and type of the biochar have sustained the level of chlorophyll contents. Under early blight stress highest chlorophyll contents (42.95 ± 0.33) were in ‘Comp + 6%GWB + PGPR’ treatment, while the lowest content level (33.61 ± 0.28) was measured in soil control in the absence of PGPR.

**Table 2:** Effect of *Alternaria solani* and PGPR on chlorophyll contents of tomato plants grown in different soil substrate compositions including compost alone and in combination with 3 and 6% of wood biochar (WB) and green waste biochar (GWB).

Treatments		Chlorophyll contents (SPAD value)
Soil − PGPR	− *A. sol*	34.39 ± 0.21^kl^
	+ *A. sol*	33.61 ± 0.28^l^
Soil + PGPR	− *A. sol*	35.65 ± 0.37^jk^
	+ *A. sol*	34.17 ± 0.32^kl^
Comp − PGPR	− *A. sol*	36.62 ± 0.23^ij^
	+ *A. sol*	35.90 ± 0.30^j^
Comp + PGPR	− *A. sol*	39.26 ± 0.20^efg^
	+ *A. sol*	38.14 ± 0.30^gh^
Comp + 3%WB − PGPR	− *A. sol*	38.10 ± 0.19^ghi^
	+ *A. sol*	37.10 ± 0.22^hij^
Comp + 3%WB + PGPR	− *A. sol*	39.32 ± 0.28^efg^
	+ *A. sol*	38.56 ± 0.34^fgh^
Comp + 6%WB − PGPR	− *A. sol*	40.02 ± 0.32^def^
	+ *A. sol*	39.00 ± 0.34^efg^
Comp + 6%WB + PGPR	− *A. sol*	40.91 ± 0.24^ cd^
	+ *A. sol*	40.00 ± 0.20^def^
Comp + 3%GWB − PGPR	− *A. sol*	41.07 ± 0.21^ cd^
	+ *A. sol*	40.09 ± 0.14^de^
Comp + 3%GWB + PGPR	− *A. sol*	42.11 ± 0.31^bc^
	+ *A. sol*	41.25 ± 0.40^ cd^
Comp + 6%GWB − PGPR	− *A. sol*	43.07 ± 0.08^ab^
	+ *A. sol*	41.97 ± 0.39^bc^
Comp + 6%GWB + PGPR	− *A. sol*	44.02 ± 0.24^a^
	+ *A. sol*	42.95 ± 0.33^ab^

Data were mean values ± standard error (n = 5) followed by different letters in the superscript suggest significant difference as per Tukey’s HSDtest (*P* ≤ 0.05).

SPAD-502—Soil Plant Analyses Development chlorophyll meter of Konica Minolta company was used.

#### Nitrogen, phosphorous and potassium contents of tomato plants

**Figure 4:**
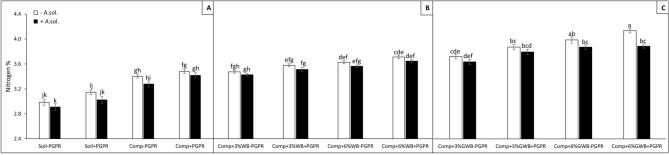
Effect of *Alternaria solani* and PGPR on percentage of nitrogen in leaf tissues of tomato raised in various soil substrate compositions including; (**A**) soil and compost (Comp) alone with (+ PGPR) and without PGPR (− PGPR), inoculated (+ *A. solani*) or un-inoculated (− *A. solani*), (**B**) compost (Comp) with 3 and 6% wood biochar (WB) with (+ PGPR) and without PGPR (− PGPR), inoculated (+ *A. solani*) or un-inoculated (− *A. solani*), (**C**) compost (Comp) with 3 and 6% Green waste biochar (GWB) with (+ PGPR) and without PGPR (− PGPR), inoculated (+ *A. solani*) or un-inoculated (− *A. solani*). All values represent mean ± SE, recorded 40 days after transplantation. Bars with different letters on the top suggest significant difference as per Tukey’s HSD test (*P* ≤ 0.05).

ANOVA analysis highlighted the significant (*P* < 0.001) effect of soil composition, PGPR and *A. solani* (Table 1) on Nitrogen (N) contents of tomato plants. Figure 4A–C demonstrated a higher percentage of nitrogen (N) contents in tomato shoots grown in both wood and green waste biochar with and/or without PGPR. Highest N contents (%) 4.11 and 3.87% were measured in ‘Comp + 6%GWB + PGPR’ treatment both in the absence and presence of *A. solani*, respectively (Fig. 4C). Tomato plants grown in wood biochar has sustained the level of N contents under disease stress as compared to their respective healthy compliments (Fig. 4B).

**Figure 5:**
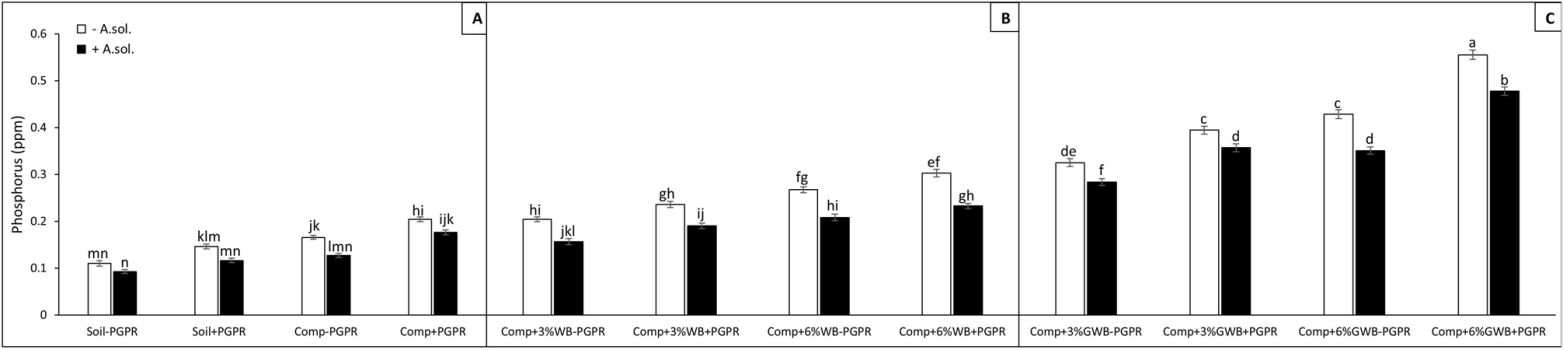
Effect of *Alternaria solani* and PGPR on phosphorus contents of leaf tissues in leaf tissues of tomato raised in various soil substrate compositions including; (**A**) soil and compost (Comp) alone with (+ PGPR) and without PGPR (− PGPR), inoculated (+ *A. solani*) or un-inoculated (− *A. solani*), (**B**) compost (Comp) with 3 and 6% wood biochar (WB) with (+ PGPR) and without PGPR (− PGPR), inoculated (+ *A. solani*) or un-inoculated (− *A. solani*), (**C**) compost (Comp) with 3 and 6% Green waste biochar (GWB) with (+ PGPR) and without PGPR (− PGPR), inoculated (+ *A. solani*) or un-inoculated (− *A. solani*). All values represent mean ± SE, recorded 40 days after transplantation. Bars with different letters on the top suggest significant difference as per Tukey’s HSD test (*P* ≤ 0.05).

In case of Phosphorous (P) contents (ppm), a significant (*P* < 0.001) interaction effect of SC × PGPR and SC × AS was observed (Table 1). The maximum significant value of P contents was recorded in plants raised in 6%GWB amended soil in association with PGPR either un-infected or infected with *A. solani* (0.56 and 0.48 ppm), respectively (Fig. 5C). Whereas, the level of P contents significantly reduced in remaining GWB amended treatments inoculated with *A. solani*, both with and without PGPR. The same trend has been recorded for P contents of plants grown in wood biochar amended soil (Fig. 5B). However, the minimum (0.09 ppm) was recorded in the *A. solani* infected plants grown in soil only without compost and biochar amendments (Fig. 5A).

**Figure 6:**
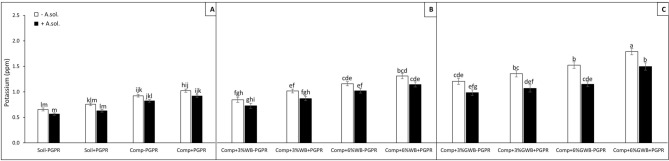
Effect of *Alternaria solani* and PGPR on potassium contents of leaf tissues in leaf tissues of tomato raised in various soil substrate compositions including; (**A**) soil and compost (Comp) alone with (+ PGPR) and without PGPR (− PGPR), inoculated (+ *A. solani*) or un-inoculated (− *A. solani*), (**B**) compost (Comp) with 3 and 6% wood biochar (WB) with (+ PGPR) and without PGPR (− PGPR), inoculated (+ *A. solani*) or un-inoculated (− *A. solani*), (**C**) compost (Comp) with 3 and 6% Green waste biochar (GWB) with (+ PGPR) and without PGPR (− PGPR), inoculated (+ *A. solani*) or un-inoculated (− *A. solani*). All values represent mean ± SE, recorded 40 days after transplantation. Bars with different letters on the top suggest significant difference as per Tukey’s HSD test (*P* ≤ 0.05).

Tomatoes infection with *A. solani* had a significant impact on lowering the potassium (K) contents (ppm) in all of the treatments (Fig. 6A–C). Data analysis revealed significant (*P* < 0.01) interactive effect between soil amendments and *A. solani* on K contents of tomato plants. In comparison with all of the treatments, plants grown in soil amended with green waste biochar depicted higher K contents, with the maximum of 1.94, 1.72 and 1.70 ppm in ‘Comp + 6%GWB + PGPR’, ‘Comp + 3%GWB − PGPR’ and *A. solani* inoculated plants in ‘Comp + 6%GWB + PGPR’ treatment, respectively (Fig. 6C). The factor PGPR also significantly (*P* < 0.001) influenced the K contents in tomatoes. However, plants grown in wood biochar (6%) amended treatments were 1.43 and 1.33 ppm, in the presence and absence of PGPR, respectively (Fig. 6B). The lowest value of K contents (0.56 ppm) was recorded in *A. solani* inoculated plants grown in soil without any compost and biochar amendment.

### *Alternaria solani* incidence, percent disease index and assessment of disease response of tomato plants

Early blight incidence, percent disease index (PDI) and tomato plant response to disease was assessed thirty 4r days after transplanting on the basis of symptoms development and severity. The incorporation of biochar in plant growth medium has a suppressive effect on *A. solani* development on tomatoes alone and in combination with PGPR (Table 3). In case of soil amendment carrying 3% GWB, an increase in disease incidence from 40 (Comp + 3%GWB-PGPR) to 60% (Comp + 3%GWB + PGPR) was observed. Whereas, PDI was significantly reduced (10.53%) in the ‘Comp + 3%GWB + PGPR’ treatment in comparison to its non-PGPR counterpart. Minimum disease incidence (20%) and PDI (20 ± 1.26) was observed in tomato plants raised in 6%GWB amended soil substrate in the presence of PGPR (Comp + 6%GWB + PGPR). Similarly, there was a reduction of 12.8 and 19.18% in the PDI, as recorded in treatment ‘Comp + PGPR’ and ‘Comp + 3%WB + PGPR’, respectively, when compared to their—PGPR counterparts.

**Table 3:** Effect of different soil substrate compositions and plant growth promoting rhizobacteria (PGPR) on disease incidence, percent disease index and disease responses of tomato plant against *Alternaria solani.*

Treatments*	DI (%)	PDI ± SE	DR
Soil − PGPR	100	80.8 ± 1.50^a^	HS
Soil + PGPR	80	80.0 ± 2.19^a^	HS
Comp − PGPR	80	68.8 ± 2.33^b^	HS
Comp + PGPR	80	60.0 ± 1.26^c^	S
Comp + 3%WB − PGPR	60	58.4 ± 2.04^c^	S
Comp + 3%WB + PGPR	60	47.2 ± 2.33^d^	S
Comp + 6%WB − PGPR	60	44.8 ± 2.33^d^	S
Comp + 6%WB + PGPR	40	42.4 ± 1.60^de^	S
Comp + 3%GWB − PGPR	40	37.6 ± 2.04^e^	MR
Comp + 3%GWB + PGPR	60	31.2 ± 1.50^f^	MR
Comp + 6%GWB − PGPR	40	23.2 ± 1.50^g^	R
Comp + 6%GWB + PGPR	20	20.0 ± 1.26^g^	R

*All treatments were inoculated with *Alternaria solani.*

*DI* disease incidence, *PDI* ± *SE* percent disease index ± standard error, *DR* disease response, *R* resistant, *MR* moderately resistant, *S* susceptible, *HS* highly susceptible.

Overall, tomato plant response to *A. solani* varied from highly susceptible (S) to resistant (R) grown in different soil substrate compositions, with the PDI values ranging between 20 and 80.8%. The plants grown in ‘Comp + 6%GWB + PGPR’ have shown ‘R’ response to early blight followed by moderately resistant (MR) response in ‘Comp + 3%GWB + PGPR’, and ‘Comp + 3%GWB − PGPR’, while susceptible (S) response in all of the wood biochar amended treatments was recorded (Table 3). Whereas, tomato plants were highly susceptible (HS) to *A. solani*, when grown in the absence of any soil amendment as well as in the treatment containing only compost (Comp-PGPR).

### In vitro effect of compost, biochar and *Bacillus subtilis* on *Alternaria solani* mycelium growth and development

In vitro toxicity of compost and biochar (WB and GWB) amended PDA media and PGPR towards *A. solani* is shown in Table 4, while the un-amended media served as a control. The lowest (1.77%) inhibition of fungal radial growth was recorded in media modified with compost. Further, no significant difference in *A. solani* mycelium growth inhibition (10.27 and 15.91%), in WB (3%) and GWB (3%) amended media, respectively. However, with the increase in the concentration of biochar i.e. WB (6%) and GWB (6%) in PDA, the efficiency of fungal radial growth inhibition was also increased. So it was observed that GWB (6%) has induced significantly higher (38.74%) *A. solani* mycelium growth inhibition, followed by the media amended with WB (6%; 29.21% inhibition) in comparison to the control. The maximum mycelia growth inhibition (55.75%) was recorded in *B. subtilis* inoculated PDA, surpassing all other treatments used in the assay.

**Table 4:** In vitro mycelium radial growth (mm) and inhibition (%) of *Alternaria solani* in control (un-amended), compost (Comp, 20%), PGPR (*Bacillus subtilis*), wood biochar (WB, 3%, 6%), green waste biochar (GWB, 3%, 6%) amended PDA.

Treatments	Radial growth (mm)	Inhibition (%)
Control	82.3 ± 0.80^a^	
Comp	80.8 ± 0.58^a^	1.77 ± 1.48^e^
WB (3%)	73.8 ± 1.16^b^	10.27 ± 2.02^d^
WB (6%)	58.2 ± 1.28^b^	29.21 ± 2.15^c^
GWB (3%)	69.2 ± 5.83^c^	15.91 ± 2.16^d^
GWB (6%)	50.4 ± 0.93^d^	38.74 ± 1.28^b^
PGPR	36.4 ± 0.51^e^	55.75 ± 0.81^a^

Given results are mean values ± standard error followed by different letters in the superscript within a column denotes significant differences according to Tukey’s HSD test (*P* ≤ 0.05).

## Discussion

Phyto-pathogens minimize the yield and deteriorate the quality of agricultural products causing significant economic losses to the agricultural entrepreneurs^60^. Excessive use of agricultural chemicals incite environmental and health issues as well as leads to the mutations in pathogenic strains rendering them resistant to existing disease management practices^61^. In this regard, emergence of multiple *A. solani* isolates^62^, with enhanced resistance against fungicides such as azoxystrobin, pyraclostrobin^63^, mancozeb and chlorothalonil^64^, ignites the need for the development of sustainable disease management strategies duly harmonized with the environment. Therefore, the current study was conducted with the aim of developing a novel, sustainable and economically viable approach to enhance crop productivity by reducing pathogen induced losses without damaging the diversity of life around^65^.

Biochar application is an ancient method to improve the soil quality, however it is only been last two decades that witnessed the keen interest of researchers towards biochar as carbon sequestration and organic plant protection agent^66^. The individual effects of compost and *B. subtilis* against EB of tomato has already been documented^36,48^. However, the response of tomatoes grown in biochar and PGPR modified soil medium against EB has not been explored earlier. To our knowledge, the results presented here for the first time elucidate the effectiveness of compost, biochar mixture together with PGPR on foliar pathogen (*A. solani*) development in tomatoes.

The different biochars with diverse compositions don’t follow uniform application rate principal to get desired response either in case of plant health improvement or disease suppression^27,67^. Similarly, in our study, not only biochar types but also their application rate influenced the EB disease incidence, severity and plant’s vegetative as well as physiological responses. We found the ineffectiveness of WB at both application rates in suppressing the disease. On the contrary GWB significantly reduced the disease at both of the application rates i.e. 3 and 6%, while being more effective at higher concentration. Zwart and Kim^68^, documented the reduction in *Phytophthora* spp. induced stem lesions in landscape tress species (*Acer rubrum* and *Quercusrubra*) grown in potting media amended with 5% biochar made from raw material of pine (*Pinus* spp*.*) origin, whereas Elad et al.^20^ found the enhanced suppression of *Botrytis cinerea* at higher application rate of biochar. In contrast with our finding, Harel et al.^28^ demonstrated the reduction in disease inhibition of foliar pathogens causing powdery mildew and grey mold on strawberry at comparatively lower biochar doses. While, Atucha and Litus^69^, documented the effectiveness of pinewood biochar at much higher application rates i.e. 10 and 20% (v/v), against replant disease in susceptible peach rootstock. Thus, effective diseases suppression and plant growth promotion rely heavily on biochar type and concentration to be applied in the potting medium.

However, there are also studies suggesting antagonistic effect of biochar on plant protection against diseases such as of maple wood bark biochar application increased the severity of *Rhizoctonia solani* in multiple plant species including crops of horticultural importance such as tomato, carrot, radish and others^70^.

The control of plant pathogens with biochar could be the result of direct toxicity to only soil-borne pathogens, however, in case of foliar pathogens the probable mode of action needs further elucidation. Earlier studies conducted on biochar effect on *Botrytis cinerea*, *Colletotrichum acutatum* and *Podosphaera apahanis* development on strawberry revealed the activation of expression of defence-related genes involved in ISR and SAR pathways^28^. Whereas, Mehari et al.^29^ reported the ISR/Jasmonic acid pathways involvement in imparting resistance in tomatoes against *B. cinerea*. So, it was proposed that systemic induced resistance plausibly the main component in suppressing foliar pathogens by biochar amendments^20^, in addition to increased/healthy plant growth. Multiple studies have repeatedly shown that composts have a suppressive effect on soil-borne diseases such as damping, root rots^71,72^ by modifying the rhizosphere and/or soil microbial profile as a whole^73^.

It is also suggested that the incorporation of PGPR with other organic soil amendments significantly contribute towards improved plant health^47^, and better protection from phyto-pathogens^74^. In a very rare study, Postma et al.^75^ reported the effectiveness of co-application of animal bone charcoal and phosphate solubilizing rhizobacteria against soil-borne disease of tomato namely, damping-off (*Pythium aphanidermatum*) and Fusarium crown and root rot (*Fusarium oxysporum* f. sp. *radicis-lycopersici*).

Our results also indicated the enhanced protection of tomatoes against *A. solani* in the presence of PGPR in biochar amended potting substrate. Addition of the biochar in soil formulate a unique environment consisting of high carbon contents, minute quantities of phenols and other organic acids with the ability to induce hormesis response^46^. While, the working efficiency of *B. subtilis* is influenced by biotic like plant genotype, microbial community and etc., as well as abiotic factors such as soil type, organic contents, and temperature^76^, clearly correlates with the findings of this study. Biochar might serve as a suitable carrier material for PGPR or bio-control agents^66,75^. Moreover, with an added advantage of enhanced survivability, multiplication and colonization in porous spaces of biochar^77^, makes it an ideal candidate for the development of bio-control formulation for commercial applications.

Therefore, the effects of PGPR must be anticipated as a result of multiple factors involving soil environment, antibiosis, induction of systemic resistance, and pathosystem under investigation^78^. However, PGPR mediated ISR is mainly considered responsible for the enhanced level of protection against foliar pathogens^52,79^. Another study attributed the *B. subtilis* induced protection from EB to the production of antioxidants and over-expression of systemic induced resistance genes^80^.

Taken together, biochar and PGPR also perform critical function in priming of host defence by inducing the activation of salicylic acid and jasmonic acid pathways^30^. Once primed, the plant can cope with the challenging pathogens more aggressively and efficiently^81^. Both biochar borne chemicals and PGPR inoculation potentiate the systemic resistance and cascade of defence related signaling events^82,83^.

Plant growth response to biochars depend upon the organic material used for the pyrolysis^46^. In this study, GWB addition in tomatoes growing medium containing compost had a significant positive impact on plant growth, when applied at 6% (v/v) application rate. Previously, She et al.^84^ found that there was an increase in tomato vegetative growth parameters at higher doses of wheat straw biochar. Whereas, Rajkovich et al.^85^ documented that the change in feedstock type produced a variable growth patterns in corn. In general, application of biochar with proper nutrient source such as compost, could have a positive influence on plant health and production^86^. Earlier, Schulz and Glaser^43^, found that the application of biochar with compost was more desirous than with the mineral fertilizers in terms of improving plant growth. Further, biochar and compost had a positive impact on soil properties and in increasing the growth of plant as reported by Safaei Khorram et al.^87^.

Moreover, the differences in plant growth in either WB and/or GWB amended potting media might be due to the differences in their nutrient retention capacity. As, the nutrients from biochars made from leaf-like material are easily accessible to the plants then the biochars obtained from woody feed stock^26,88,89^. Similarly, Hossain et al.^90^ reported the increase in tomato growth raised in soil modified with wastewater sludge biochar to enhanced nutrient retention and availability of N and P. While, Vaccari et al.^91^ described an enhanced availability of N, P and K to the tomato plants grown in biochar treated soils.

Overall, increase in growth could also be due to the additional liming impact of the biochar, thereby increasing the plant’s efficiency of nutrient utilization^92^. Our results had also revealed that the increased tomato agronomic and physiological growth parameters to the treatments with greater concentration of either WB or GWB because of their increased ability to lower the soil pH.

The additional growth promotion by PGPR, might not only be due to the nutritional or liming effect, other factors such as production of plant growth hormones, biocontrol activity and organic acids plausibly contribute to activated plant growth response. Egamberdieva et al.^54^ reported an increase in soybean (*Glycine max* L.) plant growth grown in hydrochar (2%) attributed to enhanced plant growth promoting rhizobacterial activity in the root zone. The production of Indole 3-acetic acid (IAA) by PGPR has a major share in activating plant cellular multiplication which contributes in development of a vigorous roots network. Araujo et al.^93^ also reported the production of IAA and abscisic acid by *B. subtilis* strains resulting in root growth promotion. Additionally, PGPR are also known to aid in solubilisation of unavailable form of nutrients by organic acid production^94^ thus, facilitating the transport of nutrients from rhizosphere to the plant^95^. As a result, enhanced nutrient uptake from well-established root system corresponds to increased metabolic activity as well as growth and development of above-ground plant parts^96,97^.

Parallel to the tomatoes growth response, the chlorophyll contents also responded in the same way to biochar amendment (little or no effect of WB, while positive effect of GWB) both in the presence and absence of EB stress. Previous studies also contradict in terms of the effect of biochar on photosynthetic pigments, like Akhtar et al.^98^ who described decreased chlorophyll contents of tomato plants grown in biochar, while in one of our previous studies, there was no reducing effect of biochar on chlorophyll contents^46^. In addition to enhancing nutrient solubilisation (P and K), the association of PGPR with biochar clearly enhanced and maintained the level of chlorophyll even in the presence of *A. solani*. Similarly, Danish and Zafar-ul-Hye^99^, reported significant increase in chlorophyll contents of wheat in response to the synergistic effect of PGPR and biochar. Biochar induced alterations in communication or signaling mechanisms between plant and microbes might also be responsible for the changes in PGPR response in the presence of different biochars used in variable concentrations in the soil substrate^100,101^. The mechanisms and processes involved in plant growth improvement with simultaneous protections form diseases are complex and signifies the need of further in depth analysis for complete understanding.

*Alternaria solani* can survive in the soil in the form of fungal mycelia and conidia on host debris^102^, whereas chlamydospores even in the absence of host debris^103^. So, to anticipate the direct impact of compost, biochar and *B. subtilis* on *A. solani* spores and mycelium overwintering in the field, PDA plate assay was employed^26,48^. As expected, compost has lost its antifungal property after autoclaving of the PDA, consequently minimum fungal inhibition was observed. Probably highlighting the role of the compost inhabiting microbes in suppressing the pathogenic microbes^104,105^. Although, incorporation of biochar has produced varying degree of *A. solani* mycelial growth inhibition but none of the biochars either WB or GWB were able to suppress inhibition in close proximity to 50%. Previous studies were also in agreement that disease suppression was not often lies in correspondence to the levels of in vitro toxicity of organic amendments^26,67^. *Bacillus subtilis* is known to have antagonistic effects against *A. solani*^106^. We also found greater inhibition of *A. solani* mycelial growth induced by *B. subtilis*, which could be due to the production of extracellular compounds including biosurfactants like iturin and fengycin causing antibiosis to fungal pathogens. On et al.^107^ also published about the antifungal activity of *B. subtilis* culture crude extracts against *A. solani*. The direct antifungal effect of organic amendments and PGPR, yet provide with another possibility of limiting the level of overwintering inoculum of *A. solani* in soil and plant debris. However, future experimentation will decide the faith of this assumption.

Depending upon the type of feedstock and biochar concentration in the soil substrate, two different types of biochars i.e. WB and GWB had a variable impact on plant health and early blight development in tomatoes. Based on the comparative analysis, GWB was found to be the most effective in suppressing *A. solani*, alone as well as in combination with *B. subtilis*. The combined application of biochar, compost mixture with PGPR, stimulated the rhizobacterial activity resulting in plant growth activation and disease inhibition. In the next phase, we are planning on studying genes associated with induced resistance to confirm their role in suppressing early blight in tomatoes. However, the concentration level of biochar to be used as a soil amendment is a subject deserving more research. In addition, future research activities should be focused to decipher the mechanisms behind the biochar induced resistance in tomato plants against *A. solani* as well as in other patho-systems of economic importance. In order to address the possible risks associated with biochars application on plant health, there is dire need of attention by scientific community in understanding biochemistry of the processes triggered by biochar borne chemicals.

## Materials and methods

### Isolation and characterization of *Alternaria solani*

Infected tomato plants exhibiting characteristic symptoms of early blight growing under field condition at University of the Punjab Lahore, Pakistan were identified for the fungal isolation. Standard tissue segment technique was followed^108^ to obtain *A. solani* culture on potato dextrose agar (PDA) medium (MERCK).

Subsequently, fungal mycelia from previously incubated PDA plates were shifted to freshly prepared media plates. Pure culture of the *A. solani* was obtained by re-culturing of isolated fungi via single spore technique^109^ and maintained as stock culture on Agar slants at 5 °C for future usage. Identification of pathogen was done by cultural and morphobiometric properties (Supplementary Table S1) as per Ellis^110^, and Simmons^111^. Linear growth of fungus was deliberated by measuring diameter of colonies in the same axis using transparent plastic scale in millimeter after 7 days of inoculation^112^. Fungal culture characteristics such as topography of mycelium, color and margin of colony on PDA were recorded^113^. For calculation of number of spores, a spore suspension was prepared by transferring 5 mm diameter block from media into 5 mL distilled water in a test tube and stirred with stirrer. Subsequently, sporulation was recorded by calculating mean value of spore count of three microscopic fields in one drop of spore suspension under object lens of compound microscope^112^.

### Molecular identification of *Alternaria solani*

CTAB (Cetyl Trimethyl Ammonium Bromide) method was used to extract genomic DNA of *A. solani*^114^. The ITS (Internal Transcribed Spacer) region of *A. solani* was amplified by using universal ITS primers [ITS1 forward (TCCGTAGGTGAACCTGCGG) and ITS4 reserve (TCCTCCGCTTATTGATATGC)] as described previously^115–117^. In order to further confirm the identity of *A. solani*, β-tubulin 1^116^ was amplified by using FP_tub (TCCCACTCCTTCCGCGCTGT) and RP_tub (TGTACCAATGCAAGAAAGCCTTG) as forward and reverse primers, respectively. The primers were designed using Primer 3.0 (http://bioinfo.ut.ee/primer3/), while the self-annealing of primers was checked using OligoCalc (http://biotools.nubic.northwestern.edu/OligoCalc.html). PCR reaction was performed as described^116^. PCR products were sequenced at Macrogen Inc. (Seoul, South Korea).

### *Bacillus subtilis* culture

The *Bacillus subtilis* (Genbank accession No. LC425129.1) isolate of PGPR was provided by the Microbiology lab of Institute of Agricultural Sciences (IAGS), University of the Punjab Lahore, Pakistan. The inoculum was prepared by re-culturing in nutrient broth (MERCK, USA) and incubating on shaker at 120 rpm for 36 h at 28 ± 2 °C. Afterwards, bacterial culture was centrifuged at 5,000 rpm for 10 min at 4 °C. Newly formed pallet was suspended in sterile distilled water and concentration of the bacterial suspension was adjusted at 10^8^ colony-forming unit (CFU)/mL (OD600 = 1.0) according to the Qiao et al.^118^.

### Soil substrate preparation and experiment setup

Depending upon the originating feedstock, two types of biochar, green waste biochar (GWB), produced from garden waste material and wood biochar (WB), produced from beech wood chips at pyrolysis temperature of 500 °C were used in the experiment. Both of these biochar differ substantially in structure and chemistry from each other (e.g. nitrogen contents, pH, cation exchange capacity and others) as described by Akhter et al.^46^ and Frišták et al.^119^. The compost was obtained from National Fertilizer Marketing of Govt. of Punjab, Lahore Pakistan with product name of ZameenDost (ZD) under license no. 1140. The most pertinent characteristics of biochars along with the compost are described in Table 5.

**Table 5:** Physiochemical parameters of soil, compost, wood biochar, green waste biochar.

	pH	CEC (mmol 100/mL)	Density (kg/L)	EC (mS/cm)	OM (%)	SA (m^2^/g)	AC (%)	C (%)	H (%)	P (%)	N (%)	K (%)	Cd (mg/kg)	Cu (mg/kg)	Zn (mg/kg)
Soil	8.01	–	0.75	1.02	0.604	–	–	1.07	–	2.10	0.07	1.87	–	82.80	42.10
Comp	7.18	–	0.63	1.32	17.20	–	–	28.54	–	0.40	1.20	0.55	0.03	73	462
WB	8.78	9.83	0.36	0.54	–	27.24	15.20	80.30	1.60	–	0.40	–	< 2	16	93
GWB	9.03	12.85	0.34	1.67	–	31.54	19.30	79.78	1.59	–	0.35	–	< 2	21	95

*CEC* cation-exchange capacity, *EC* electrical conductivity, *OM* organic matter, *SA* surface area, *AC* ash contents, *Comp* compost, *WB* wood biochar, *GWB* green waste biochar.

– Parameters were not analyzed.

Sterilized sandy loam soil containing 5.5% clay (< 2 mm), 42.7% silt (> 2 mm), 51.8% sand (> 63 mm), having bulk density 1.20 g/cm^3^ (PCRWR), was collected from experimental fields of IAGS (0 to 15 cm depth). It was used as basic material to make different compositions of potting mixture with compost (Comp) (20% v/v) and/or WB (3 and 6% v/v), GWB (3 and 6% v/v) for the plant cultivation.

The experiment set up was comprised of following treatments: (i) soil, (ii) Comp, (iii) Comp + 3%WB, (iv) Comp + 6%WB, (v) Comp + 3%GWB, (vi) Comp + 6%GWB, with (+ PGPR) and/or without PGPR (− PGPR). The treatments were either inoculated with *A. solani* (+ *A. solani*) or free from fungal inoculm (− *A. solani*). The experiments were conducted twice, while each treatment consisted of five replicates with each replicate comprised of a pot (Volume: 2 L, 15.5 cm height × 14 cm width) containing a plant.

### Tomato plant propagation

Tomato seeds (*Solanum lycopersicum* L. cv. Rio grande) were surface sterilized with 3% NaOCl solution by soaking for 10 min and then rinsed thrice with double distilled water to wash off the chemical. Seeds were sown in trays containing double autoclaved potting mixture comprising of peat, perlite (Gro-Sure Westland Horticulture Cambridgeshire UK) and compost (ZD) (1:1:1, v/v/v). The trays were then incubated in a growth chamber at 24 °C with a 15/9 h light/dark photoperiod (light intensity 296 µmol m^−2^ s^−1^). The trays were irrigated regularly with tap water. After four weeks, tomato seedling were reached at 1–3 true leaf stage and transferred from trays to pots containing potting mixture as described in previous section. For PGPR inoculated treatments, the roots of tomato seedlings were dipped in PGPR suspension of 10^8^ CFU/mL concentrations for 1 min before transplantation and 10 mL suspension was applied in the soil around the rhizosphere ten days after transplantation^120^.

For inoculation, the *A. solani* was cultured on Petri dishes containing PDA (MERCK, USA) and stored for 3 weeks at 25 °C in the dark in DNP-9022 incubator. To make suspension for inoculation, conidia were harvested by flooding the Alternaria culture plates with autoclaved water and gently scraping the colony surface with spatula. Next, the suspension was filtered using four layers of cheesecloth (50 µm). Final concentration of conidial suspension was determined and adjusted at 1 × 10^6^condia/mL with hemocytometer^121^.

After 14 days of transplantation, conidial suspension of *A. solani* (10^6^ conidia/mL) was inoculated on plants^80^. Conidial suspensions were sprayed gently on tomato leaves in the evening by following direct spray inoculation method using manual sprayer (Nozzle size = 0.8 mm)^122^. The inoculated tomato plants were sprayed with sterilized water for 2 days to maintain the required humidity (approx. 70%) for disease development. The plants were maintained in a greenhouse in randomized manner as per all the recommended practices for cultivation of tomato to raise a good crop^123^.

### Plant growth assessment

The plants were harvested 40 days after transplantation by gently uprooting followed by washing the roots under running tap water to record growth parameters such as plant height, root and shoot dry weight as per Awan et al.^11^. Plant height was measured from base of stem to top of plant. Further, to calculate the dry weights, both roots and shoots were cut separated and dried in an air circulation oven at 60 °C for 7–10 days unless no change in weight was recorded.

### Plant physiological parameters assessment

#### Chlorophyll contents determination

Two days prior to harvesting, a portable chlorophyll meter SPAD-502 (SPAD-502-Soil–Plant Analyses Development chlorophyll meter, Konica Minolta) was used to measure the chlorophyll contents of leaves (3rd pair from the top)^124,125^. To minimize the chances of errors, each obtained value represents an average of three readings.

#### Nitrogen, phosphorus and potassium contents determination

For NPK quantification, tomato leaves from each treatment were obtained and dried in oven for 4 consecutive days at 65 °C and ground into fine powder using pestle and mortar. Total nitrogen in leaf samples was assessed through Kjeldahl digestion method as described by Islam^126^, using automatic Kjeldahl apparatus (BD40, LACHAT, US). To obtain the mean value of percent nitrogen content, five samples from each treatment were digested. For the purpose, 0.1 g of powdered sample was digested with 4 mL of H_2_SO_4_ at 420 °C for 1 h. To attain optimum results, K_2_SO_4_ and CuSO_4_ were added as catalysts at a ratio of 9:1^97,127^.

For the estimation of total P and K, two 0.5 g samples were prepared by wet digestion method as explained by Uddin et al.^128^ and Hseu^129^. Further, the concentration of P and K in digested samples were determined by following spectrophotometric vanadium phosphormolybdate method^97,130^ by using AA spectrophotometer (AA-6200, Shimadzu US) at 420 nm and flame photometric method^130,131^ by using Industrial Flame Photometer (PFP7, Jenway, UK), respectively. The concentrations in samples were determined by comparing with standard curve^132^.

### Disease assessment

Early blight disease severity was recorded visually 20 days after inoculation on the basis of area of leaves covered by early blight symptoms using zero to five disease rating scale (Table 6) followed by Akhtar et al.^117^. Further, percent disease index (PDI) was calculated by following formula described by Pandey et al.^133^ and Yadav et al.^134^:

$${\text{Percent }}\;{\text{Disease}}\;{\text{ Index (PDI)}} = \frac{{{\text{Sum }}\;{\text{of }}\;{\text{all }}\;{\text{rating }} \times {100}}}{{{\text{Total }}\;{\text{no}}{. }\;{\text{of }}\;{\text{observations }} \times {\text{ Maximum }}\;{\text{rating}}\;{\text{ grade}}}}.$$

**Table 6:** Disease rating scale for early blight disease on leaves of tomato plant.

Rating	Description of symptoms	PDI	DR
0	No visible symptoms/free from infection	0	I
1	1–2 spots confined to lower leaves covering 1–10% of leaf surface	0.01–10	HR
2	Few isolated spots covering 11–25% of leaf surface	10.01–25	R
3	Many spots covering 26–40% of leaf surface	25.01–40	MR
4	Many spots covering 41–60% of leaf surface	40.01–60	S
5	Many spots covering more than 60% of leaf surface	> 60.01	HS

*PDI* percent disease index, *DR* disease response, *I* immune, *HR* highly resistant, *R* resistant, *MR* moderately resistant, *S* susceptible, *HS* highly susceptible.

Disease incidence of early blight was calculated 20 days after inoculation as percentage of diseased plants in treatment following formula by Awan et al.^11,80^:

$${\text{Disease }}\;{\text{incidence (\% ) }} = { }\frac{{{\text{Number }}\;{\text{of }}\;{\text{diseased }}\;{\text{plants}}}}{{{\text{Total }}\;{\text{number }}\;{\text{of}}\;{\text{ plants}}}}{ } \times { 100}{\text{.}}$$

### In vitro toxicity of compost, biochar and *Bacillus subtilis* in PDA plate to *Alternaria solani*

The inhibitory impact of compost, WB and GWB on growth and inhibition of *A. solani* was studied in vitro on PDA plates. Both types of biochar i.e. WB and GWB, and compost were sieved through 100 μm sieve before adding to PDA^67^. The growth media was amended with compost (20%, w:v) and different concentrations of WB and GWB (3 and 6% (w:v), each) before autoclaving. Afterwards, growth media was poured into Petri-dishes (90 mm) and kept at room temperature till solidification. Subsequently, six mm diameter agar plugs of actively growing parts of fungal culture (5 days old) were obtained with sterile cork borer and placed at the center of dishes. While antifungal potential of PGPR was determined according to dual culture method by inoculating the PDA plates with *B. subtilis* close to the edges of petri-dishes^48^. The inoculated Petri-dishes were incubated at 23 ± 2 °C for 6 days. The fungal radial growth (mm) for each treatment was calculated by averaging colony diameter of five randomly arranged replicates. The percentage inhibition of fungal radial growth in different media including control (C, un-amended) and amended (A) was determined according to Bekker et al.^135^ using following formula:

$$\left( {\text{Percentage inhibition}} \right){ } = { }\frac{{{\text{C }} - {\text{ A}}}}{{\text{C}}}{ } \times { 100}{\text{.}}$$

### Statistical analysis

The data analysis was carried out using Statistix 8.1 software (Statistix, USA). Percentage data were transformed before analysis. While the pooled data of the experimental repeats were used for the analysis. The data were subject to three-way analysis of variance (ANOVA) with (i) soil substrate compositions including compost alone and in combination with wood and green waste biochar; (ii) PGPR and (iii) *A. solani* as main factors. The means were compared by applying Tukey’s HSD test at *P* ≤ 0.05 level of probability.

## Supplementary information

Supplementary information.

## Data Availability

The datasets generated during and/or analyzed during the current study are available from the corresponding author on reasonable request.
