# Supplementary material for: Role of biochar, compost and plant growth promoting rhizobacteria in the management of tomato early blight disease
Source: Sci Rep. 2021 Mar 17;11:6092. doi: 10.1038/s41598-021-85633-4 (PMC7971063; doi:10.1038/s41598-021-85633-4)
Supplement: Supplementary file 1 — Supplementary information. [file 41598_2021_85633_MOESM1_ESM.pdf]

Role of biochar, compost and plant growth promoting rhizobacteria in the management of tomato early blight disease

Mujahid Rasool[1], Adnan Akhter[1*], Gerhard Soja[2,3], Muhammad Saleem Haider[1]

[1] Institute of Agricultural Sciences, University of the Punjab, Lahore, P.O Box: 54590 Pakistan.

[2]Department of Health and Environment, Austrian Institute of Technology, Tulln, Austria.

[3]Institute for Chemical and Energy Engineering, University of Natural Resources and Life Sciences Vienna, Austria

*Correspondence to: adnanakhter.iags@pu.edu.pk

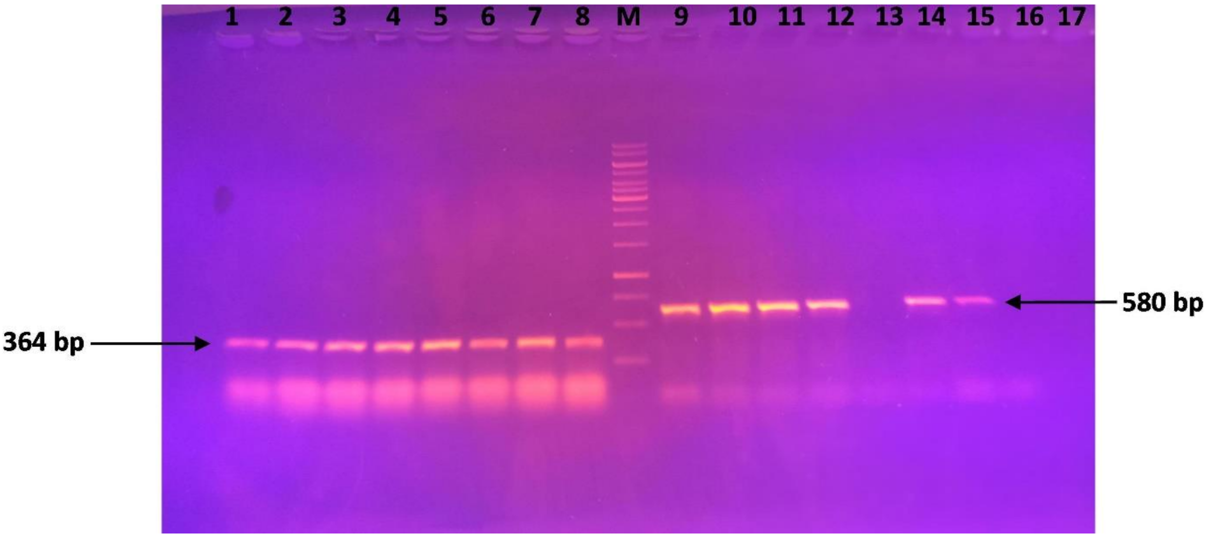

Supplementary Figure S1: Agarose gel electrophoresis of PCR products of ITS region (580 bp) and β-tubulin 1 (364 bp) as shown in lane 1-8 and 9-12, 14 and 15, respectively, while lane 16 and 17 are negative controls. Lane M represents GeneRuler 1 kb DNA ladder.

Supplementary Table S1: Morphological and microscopic characteristics of *Alternaria solani.*

Features	Description
**Colony**	Dark brown in color
**Mycelia**	Smooth growth with irregular margins
**Topography**	Aerial mycelium
**Mean Radial Growth**	88.65 mm
**Conidiophores**	Single or in groups, straight or flexuous and brown to olivaceous brown
**Conidia**	Solitary straight or slightly flexuous oblong or ellipsoidal, 150-300 µm in length, 13-20 µm thick with 6-9 transverse and 0 or few (1 to 4) longitudinal septa
**Sporulation**	21-30/Microscopic field

GenBank accession no. MT899419

***Alternaria solani* isolate MR-10 18S unit ribosomal RNA gene, partial sequence; internal transcribed spacer 1, 5.8S ribosomal RNA gene, and internal transcribed spacer 2, complete sequence; and 28S ribosomal RNA gene, partial sequence**

TCCGTAGGTGAACCTGCGGAGGGATCATTACACAAATATGAAGGCGGGCTGGCACCTCCCGGGGTGGCCAGC
CTTGCTGAATTATTTCACCCGTGTCTTTTGCGTACTTCTTGTTTCCTTGGTGGGCTCGCCCACCACAAGGACCAA
CCCATAAACCTTTTTGCAATGGCAATCAGCGTCAGTAACAATGTAATAATTTACAACTTTCAACAACGGATCTC
TTGGTTCTGGCACCGATGAAGAACGCAGCGAAATGCGATAAGTAGTGTGAATTGCAGAATTCAGTGAATCAT
CGAATCTTTGAACGCACATTGCGCCCTTTGGTATTCCAAAGGGCATGCCTGTTCGAGCGTCATTTGTACCCTCA
AGCTTTGCTTGGTGTTGGGCGTCTTTTTGTCTCCCCTTGCGGGAGACTCGCCTTAAAGTCATTGGCAGCCGGCC
TACTGGTTTCGGAGCGCAGCACAAGTCGCGCTCTCTTCCAGCCCCAAGGTCTAGCATCCACCAAGCCTTTTTTT
TCAACTTTTGACCTCGGATCAGGTAGGGATACCCGCTGAACTTAAGCATATCAATAAGCGGAGGA

GenBank accession no. MT899420

***Alternaria solani* isolate MR-10 beta-tubulin 1 gene, partial CDS**

TCCCACTCCTTCCGCGCTGTCACTGTTCCCGAGCTCACCCAGCAGATGTTCGACCCCAAGAACATGATGGCTGC
TTCCGACTTCCGCAACGGTCGCTACCTGACCTGCACGGCCTACTTCCGCGGTAAGGTCTCCATGAAGGAGGTC
GAGGACCAGATGCGCAACGTCCAGAACAAGAACTCCTCATACTTTGTTGAGTGGATCCCCAACAACGTCCAGA
CCGCCCTCTGCTCCATCCCTCCGCGCGGCCTGAAGATGTCCTCCACCTTTGTCGGTAACTCCACCTCCATCCAG
GAGCTGTTCAAGCGTGTCGGTGACCAGTTCACTGCCATGTTCAGGCGCAAGGCTTTCTTGCATTGGTACA
